# The Pathologic and Genetic Characteristics of Extranodal NK/T-Cell Lymphoma

**DOI:** 10.3390/life12010073

**Published:** 2022-01-05

**Authors:** Hyunsung Kim, Young Hyeh Ko

**Affiliations:** 1 Department of Pathology, Hanyang University College of Medicine, Seoul 04763, Korea; hhnt5841@gmail.com; 2 Department of Pathology, Guro Hospital Korea University, Seoul 08308, Korea

**Keywords:** extranodal NK/T-cell lymphoma, pathology, genetics, review

## Abstract

Extranodal NK/T-cell lymphoma is a neoplasm of NK cells or cytotoxic T cells presenting in extranodal sites, most often in the nasal cavity. The typical immunophenotypes are cCD3+, sCD3−, CD4−, CD5−, CD8−, CD16−, and CD56+ with the expression of cytotoxic molecules. Tumor subsets express NK cell receptors, CD95/CD95L, CD30, MYC, and PDL1. Virtually all the tumor cells harbor the EBV genome, which plays a key role in lymphomagenesis as an epigenetic driver. EBV-encoded oncoproteins modulate the host-cell epigenetic machinery, reprogramming the viral and host epigenomes using host epigenetic modifiers. NGS analysis revealed the mutational landscape of ENKTL, predominantly involving the JAK–STAT pathway, epigenetic modifications, the RNA helicase family, the RAS/MAP kinase pathway, and tumor suppressors, which indicate an important role of these pathways and this group of genes in the lymphomagenesis of ENKTL. Recently, three molecular subtypes were proposed, the tumor-suppressor/immune-modulator (TSIM), MGA-BRDT (MB), and HDAC9-EP300-ARID1A (HEA) subtypes, and they are well-correlated with the cell of origin, EBV pattern, genomic alterations, and clinical outcomes. A future investigation into the function and interaction of discovered genes would be very helpful for better understanding the molecular pathogenesis of ENKTL and establishing better treatment strategies.

## 1. Introduction

Extranodal NK/T-cell lymphoma (ENKTL) is an EBV-associated NK or cytotoxic T-cell lymphoma occurring at the extranodal site, predominantly in the nasal region [1]. This entity was first described by McBride P. in 1897 as a destructive lesion with necrotic granuloma of the midface as “ulceronecrotic proliferative lesions of the upper airways” [2]. Because of its characteristic clinical and histological presentations, ENKTL has various names, including “lethal midline granuloma”, “rhinitis gangrenosa progressiva”, “polymorphic reticulosis”, “reticulum cell sarcoma”, “midline malignant reticulosis”, “angiocentric immunoproliferative lesions”, and “angiocentric lymphoma” [3,4,5,6,7,8]. The nature of this entity was unknown, and the clinical course was aggressive. Most patients were treated with surgery, antibiotics, antifungal agents, steroids, and even chemotherapy and radiation therapy, without success [9]. With the application of immunophenotypic assays and in situ hybridization technology, “lethal midline granuloma” was recognized as a nasal T-cell lymphoma [10] that harbored EBV DNA and oncogenic proteins in tumor cells [11]. This tumor expressed many T-cell markers, while the TCR gene rearrangement was germline, so there was doubt as to whether the tumor-cell lineage was NK or T cell. In 1994, Suzumiya et al. reported that most of the tumor cells of this entity originated from an NK cell using immunohistochemistry and Southern blotting [12]. The Revised European–American Lymphoma (REAL) classification, published in the same year, first included this disease as a distinct subtype of malignant lymphoma, as “angiocentric lymphoma” [13]. A workshop to compare T-cell lymphomas in Asian and Western countries held in 1994 concluded that nasal T-/NK cell lymphoma, also called angiocentric lymphoma, was a distinct clinicopathologic entity, and virtually all cases of nasal T-/NK cell lymphoma are positive for EBV. The workshop proposed the terms nasal T-/NK cell lymphoma for midline facial lesions, and nasal-type T-/NK cell lymphomas for tumors in another anatomic site [14]. Lastly, the 2001 WHO classification named this entity extranodal NK/T-cell lymphoma, nasal type, which remained the same in the most recent 2017 version [1,15].

## 2. Histomorphology

**Figure 1:**
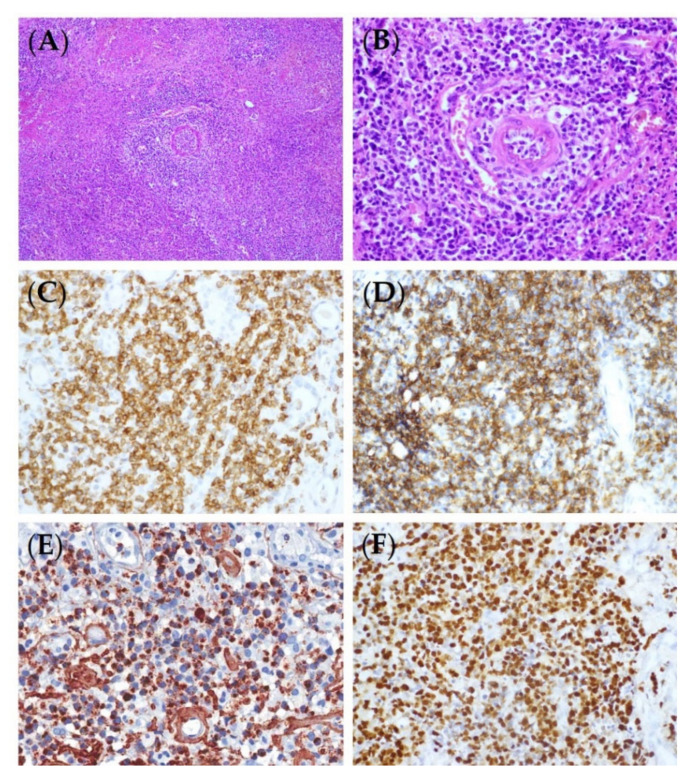
Representative microscopic and immunohistochemical features of ENKTL. (**A**) Angiocentric and angiodestructive lymphoid infiltrates in necrotic background (H&E, ×200). (**B**) The tumor cells are intermediately sized and have irregular nuclei (H&E, ×400). The tumor cells are positive for CD3 (**C**), CD56 (**D**), granzyme B (**E**), and EBER (**F**) (immunohistochemistry and in situ hybridization, ×400).

**Figure 2:**
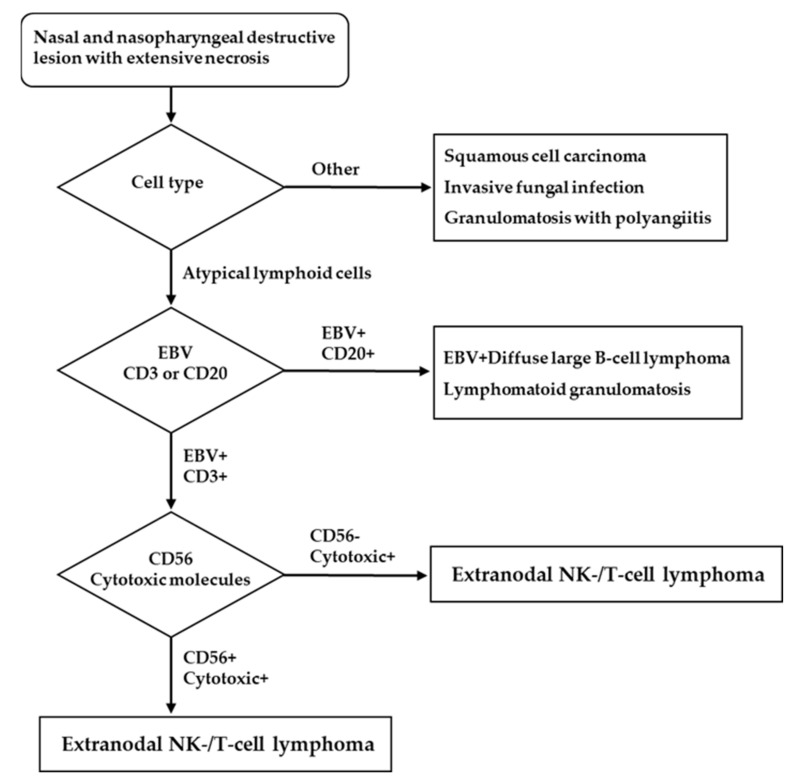
Diagnostic flowchart for ENKTL.

The histologic findings for ENKTL are similar irrespective of whether a lesion is nasal or extranasal [1]. They are characterized by diffuse and permeative lymphocytic proliferation with an angiocentric and angiodestructive growth pattern, as well as fibrinoid changes (Figure 1) [16]. The cytomorphologic spectrum, including the cellular size and grade of a pleomorphism, is very broad [17]. Most often, ENKTL is composed of medium-sized cells with focal transformed cells or mixed small and large cells [18]. Cells can have irregular nuclear contours, inconspicuous nucleoli, and granular chromatin [17]. The cytoplasm is pale to clear and moderate in amount. Mitoses are numerous, and apoptotic bodies and karyorrhexis are commonly seen [1]. Azurophilic granules can be seen in Giemsa-stained touch preparations [1]. The presence of necrosis and the admixture of inflammatory cells, particularly in small- or mixed-cell predominant cases, make it difficult to distinguish ENKTL from an inflammatory process [19]. A flowchart for ENKTL diagnosis is shown in Figure 2.

## 3. Immunophenotype

The typical phenotype of ENKTL is CD2+, surface CD3−, cytoplasmic CD3ε+, CD4−, CD5−, CD8−, CD56+, and cytotoxic markers (granzyme B, TIA-1, and perforin) (+) (Figure 1) [1] but not all cases show this phenotype. The rates of expression for the markers reported in the studies conducted using large cohorts were CD2 (93%), cCD3 (84%), CD4 (10%), CD5 (27%), CD8 (22%), CD56 (76%), granzyme B (83%), TIA1 (90%), and perforin (86%) [20,21,22]. CD56 is a useful, but not specific, marker for NK cells, and the majority of ENKTLs are positive for it, though they can be negative, especially in T-lineage ENKTLs. To diagnose a CD56-negative ENKTL as ENKTL, it must be positive for a cytotoxic molecule and EBV. A CD56-negative ENKTL has a clinical manifestation similar to that of CD56-positive cases [22]. Other NK cell markers, such as CD16 or CD57, are usually negative. NK cell receptors (NKRs), including the immunoglobulin superfamily (KIRs, killer immunoglobulin-like receptors) and C-type lectin-like family (CD94/NKG2), are expressed in a subset of ENKTLs [23]. Although it is not as specific as TCR rearrangement for determining clonality, the uniform expression of several KIRs can hint at clonal proliferation. A CD94-positive phenotype is consistent with that of a mature NK cell and implies better prognosis [24]. B cell markers, including CD20, CD79a, PAX5, and Oct2, are generally negative. The aberrant expression of CD20, CD79a, and OCT2 in ENKTL was reported in some studies, and its clinical significance is controversial [25,26,27,28]. CD95 (Fas)/CD95 ligands (CD95L) are frequently expressed, and their expression is associated with the tissue damage and necrosis seen in ENKTL [29]. MYC is expressed in 50% of ENKTLs [30] and associated with a poor treatment response and prognosis. MYC gene rearrangement is not correlated with protein expression [30,31]. More than 30% of ENKTLs are positive for CD30, whose expression is significantly higher in the extranasal type than in the nasal type [32]. The phosphatase and tensin homolog (PTEN) is the buffering molecule of the PI3K–Akt pathway [33]. Loss of PTEN expression was observed in 68% of ENKTLs, and higher PTEN expression levels significantly correlated with better treatment outcomes [34]. Programmed cell death-1 (PD-1)/PD-1 ligand 1 (PD-L1) blockade has revolutionized cancer immunotherapy, and many clinical trials are underway to evaluate its efficacy for ENKTL [35]. There is still a lack of standardized expression cutoffs for predicting the response to PD-1/PD-L1 inhibitors [36]. PD-1 is rarely expressed in ENKTL tissues (0–1.3%), whereas PD-L1 is frequently expressed in ENKTL tissues (56–79.7%) [37,38,39]. PD-L1 expression is a favorable prognostic marker in ENKTLs at advanced stages and has been associated with a low international prognostic index [37,39].

## 4. Cell of Origin

ENKTL is a tumor of either NK cell or cytotoxic T cell lineage. T and NK cells originate from the same lymphoid progenitor cells and develop into different cell lineages with the rearrangement of TCR gene [40]. Because T and NK cells share the expression of some T and NK cell markers, the cell lineage cannot be distinguished only by immunophenotype; instead, it is determined by either the TCR gene rearrangement or the expression of the TCR antigen. An early study applying Southern blot hybridization revealed T cell clonality only in a small subset (6.4%) of ENKTLs [41,42,43], indicating that most ENKTL is of NK cell lineage. Conventional polymerase chain reaction (PCR) analyses for TCR gene rearrangement exposed variable T cell clonality in 0 to 26.2% of ENKTLs [20,44,45,46,47]. BIOMED-2 multiplex PCR has a higher sensitivity when applying all primer sets. TCR gene rearrangement was detected using the BIOMED-2 method in 0–40% of ENKTLs [48,49,50]. TCR antigens are expressed in 30% of ENKTLs, and half of the ENKTLs with TCR gene rearrangement do not show TCR protein expression [50,51]. The restriction of the killer-cell immunoglobulin-like receptor (KIR) repertoire signifies monoclonal NK cell proliferation [52]. ENKTLs with restricted KIR repertoires usually lack TCR gene rearrangements, express other NKRs, and are positive for CD56, a phenotype indicating an NK cell lineage [24,52]. Taken together, about 60% of ENKTL are NK lineage, and T cell lineage tumors have a low TCR protein expression rate. The clinical features and therapeutic responses of T and NK cell lineage ENKTLs are similar [50,53].

## 5. Other EBV-Positive T- or NK Cell Lymphomas

The EBV-positive mature T/NK cell malignancies described in the 2017 WHO classification include ENKTL, EBV-positive T-cell lymphoma of childhood, aggressive NK cell leukemia (ANKL), and EBV-positive nodal T-/NK cell lymphoma (Table 1) [1,54,55].

Aggressive NK cell leukemia (ANKL) is a rare systemic lymphoproliferative disease of NK cells characterized by an association with EBV, prevalence in Asia, and aggressive clinical course [1]. ANKL is recognized as a malignancy of young to middle-aged adults, and ANKL, transformed from an EBV lymphoproliferative disease of childhood, particularly occurs at younger ages [56]. ANKL can be diagnosed based on the clinical features, involved site, and cellular characteristics [1]. Patients present with fever, a leukemic blood picture, hepatosplenomegaly, disseminated intravascular coagulation, and a progressive clinical course [1]. Commonly involved sites are the bone marrow, peripheral blood, liver, and spleen. The involved bone marrow shows diffuse or patchy destructive proliferation [1]. Leukemic cells show a wide range of morphology, from normal large granular lymphocytes to highly atypical features, with enlarged nuclei, irregular nuclear folding, and distinct nuclei [1]. The tumor cells show an immunophenotype identical to that of ENKTL except for the more frequent expression (75%) of CD16 compared to that of ENKTL (22%) [57]. Because of such similarity in the cellular lineages and immunophenotypes, it is often difficult to distinguish ANKL from advanced-stage ENKTL [58]. A recent study reported positivity for c-MYC (6/8) and EBER (9/12), as well as p53 overexpression in ANKL [59]. Earlier studies reported the genetic differences between ANKL and ENKTL.

Nodal T-/NK cell lymphoma is a primary nodal EBV-positive cytotoxic T- or, uncommonly, NK cell lymphoma without nasal involvement [54,55]; it is described as an EBV-positive variant of peripheral T-cell lymphoma in the 2017 WHO classification. It is a systemic disease with poor outcomes. Most patients are adults, some in immune-compromised states, presenting with multiple lymphadenopathy, hepatosplenomegaly, B symptoms, and advanced stages [54]. The histologic findings are variable, monomorphic or polymorphic and usually show high-grade morphology [54]. The neoplastic cells are positive for T cell markers and cytotoxic granules. The typical phenotype is EBV-positive, CD8-positive, CD56-negative, cytotoxic αβT cells. Of the cases, 0–13% comprise γδT cells. The expression of CD56 is infrequent.

Intravascular lymphoma is rare non-Hodgkin’s lymphoma characterized by the exclusively intraluminal growth of neoplastic lymphoid cells within blood vessels [60]. Most cases of intravascular lymphoma express a B-cell phenotype, and only 22 cases with an NK/T-cell phenotype have been reported [61,62,63,64,65]. In the current WHO classification, intravascular lymphoma of the NK/T-cell phenotype is not yet recognized as a distinct entity. The geographic distribution of intravascular NK/T-cell lymphoma (IVNKTL) is similar to that of ENKTL, with a high incidence in Asia and Latin America [61,62,63,64,65]. Patients with IVNKTCL range in age from 18 to 84 years, with a male-to-female ratio of 1.2:1 [61,62,63,64,65]. The histologic findings of IVNKTCL are characterized by aggregates of large atypical cells occupying the lumina of small to medium sized blood vessels [61,62,63,64,65]. The neoplastic cells are positive for cCD3ε, CD56, and EBV and show a cytotoxic T-cell phenotype [61,62,63,64,65]. Clinically, IVNKTCL is an aggressive disease with a mostly limited response to treatment and a poor prognosis [61,62,63,64,65]. A molecular study using whole-exome and RNA sequencing on two cases of IVNKTCL revealed frequent mutations of epigenetic regulator genes [65].

## 6. Mutational Landscape of Extranodal NK/T-Cell Lymphoma

When the entity of ENKTL was introduced in the REAL classification in 1994, studies to explore the genetic alterations in ENKTL were delayed because adequate tissue for analysis was difficult to obtain due to extensive tumor necrosis and the small size of the nasal biopsy. In 1997, cytogenetic analysis by Wong K.F. et al. on recurrent deletion at 6q21–q25 in one nasal, one extranasal, and one leukemic form of NK/T-cell lymphoma provided clues for tracking genetic abnormalities in ENKTL [66]. The subsequent development of comparative genomic hybridization (CGH) and array CGH (aCGH) technology, and loss of heterozygosity (LOH) analysis allowed for uncovering recurrent alterations in ENKTL, including gains at chromosomes 1p, 6p, 11q, 12q, 17q, 20q, and Xp, and losses at 6q, 11q, 13q, and 17p. Particularly consistent DNA losses at 6q13–q14, 6q21–q23, 6q16.1–q27, 6q24.3, 6q25.3, 6q27, 6q21–q25, 6q16–22, and 6q25.2–q25.3 suggest the presence of tumor-suppressor genes in these regions [67,68,69,70,71,72]. Of these, five candidate tumor-suppressor genes, including *FOXO3*, *HACE1*, *PRDM1*, *ATG5,* and *AIM1,* were identified in the minimal region in del 6q21 [73,74]. The tumor-suppressive function of *FOXO3* and *PRDM1* in NK cell neoplasms was proven by genomic and functional analyses [75,76,77]. FOXO3 belongs to the O subclass of the forkhead family of transcription factors, and its expression is downregulated in most instances of NK cell neoplasia. The re-expression of FOXO3 inhibits NK cell growth through the induction of apoptosis and cell-cycle arrest [75,76]. PRDM1 is a pleiotropic repressive transcription factor that is essential for the terminal differentiation of B cells, but it also plays a pivotal role in the regulation of NK cell activation and maturation. In a PRDM1-knockout cell model, the loss of PRDM1 shifted NK cells toward proliferation and survival, rather than the performance of their normal functions [77]. *ATG5*, a gene essential for autophagy [78], and AIM1, a gene implicated in melanoma [79], showing generally low expression in ENKTL [74], appear to play a tumor-suppressor role, although the exact function in ENKTL is not clear. The loss of 17p13, on which TP53 is located, has frequently been identified by CGH and LOH studies [70,72,74]. The P53 mutation was observed by Sanger sequencing in 31–63% of cases in an Asian population [80,81,82]. ATR is a key regulator of the DNA damage response and causes genomic instability that promotes malignant transformation in 25% of ENKTLs [83].

**Figure 3:**
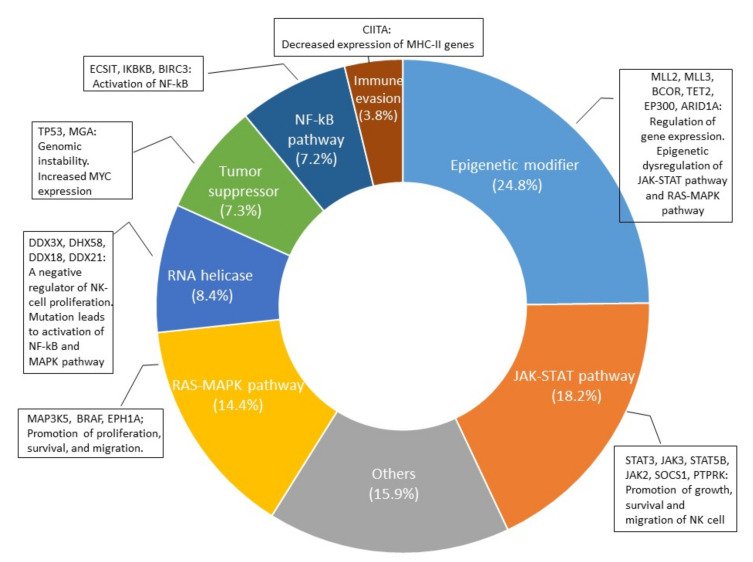
Distribution of functional groups of variants reported by nine NGS studies [84,85,86,87,88,89,90,91,92] and biologic effect of mutation.

**Figure 4:**
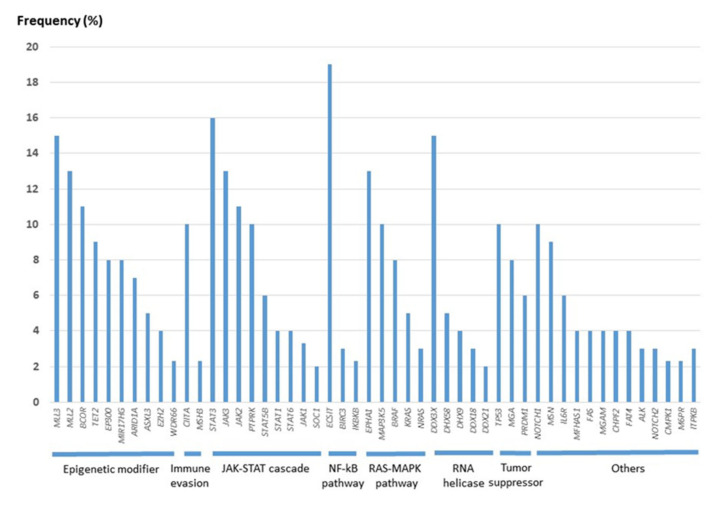
Frequency of mutation in extranodal NK/T-cell lymphoma reported by nine NGS studies [84,85,86,87,88,89,90,91,92].

The development of the NGS technique has accelerated the study of genetic mutations in NK/T-cell lymphoma and aided in examining the genomic landscape from the analysis of individual genes. In 2012, the first data from high-throughput next-generation sequencing analysis came from a Singaporean group [82]. Since then, nine papers have been published, and the multi-omic analysis of these data has expanded our knowledge on the molecular pathogenesis of ENKTL [84,85,86,87,88,89,90,91,92]. As in EBV-positive diffuse large B-cell lymphoma [93], the tumor mutational burden in ENKTL is remarkably lower than that in other aggressive lymphomas but similar to that in EBV-positive nasopharyngeal carcinoma and gastric carcinoma [91,94,95], supporting the important role of EBV in the pathogenesis of EBV-positive neoplasms. The frequency of each gene mutation is slightly different according to studies (Supplementary Table S1), but most studies share the mutation of genes involved in the JAK/STAT pathway, epigenetic modification, the RNA helicase family, the RAS/MAP kinase pathway, and tumor suppressors, which indicate an important role of these genes in the lymphomagenesis of ENKTL. When the cases included in the nine studies were combined, the gene of the epigenetic modifier group had the highest frequency of mutation, followed by the gene of the JAK/STAT pathway (Figure 3). The mutation frequency of each gene is shown in Figure 4.

- A. JAK/STAT Pathway

Janus kinase/signal transducer and activator of transcription (JAK/STAT) signaling is crucial for NK cell development and maturation [96]. When a cytokine binds to its transmembrane receptor, receptor-associated JAKs and phosphorylate STAT proteins are activated. Activated STAT proteins translocate to the nucleus, where they function as transcriptional activators of target genes [97]. According to next-generation sequencing analysis, genes of the JAK/STAT signaling pathway are mainly altered in ENKTLs of the NK lineage [91]. STAT3 mutations are clustered on the SH domain, which is critical for STAT activation [89], and are the most common mutation in genes involving the JAK/STAT signaling pathway, found in 83 of 526 cases (15.8%; range: 3–27%) [84,85,86,87,88,89,90,91,92]. The activating JAK3 mutation is clustered in the JH2 pseudokinase domain, which has no kinase activity and is thought to interact with STAT and negatively regulate the kinase activity of the JH1 kinase domain [98]. The JAK3 mutation was reported to occur in 30 of 224 ENKTL cases (13.4%; range: 0–35.4%) [84,86,90,91,99,100,101]. An in vitro study showed that the constitutive activation of the JAK3/STAT3 pathway plays a major role in ENKTL cell growth and survival, and in the invasive phenotype [101]. In addition to these genes, STAT5B and JAK2 were affected by mutations in 17 of 293 cases (5.8%) [88,89,91,92] and 11 of 100 cases (11%) [91], respectively. Suppressors of cytokine signaling (SOCS) proteins inhibit the JAK/STAT signaling cascade by suppressing JAK kinase activity, which appears to be a classic negative-feedback loop [102]. SOCS1 mutation is uncommon [87] but seems to contribute to the dysregulation of the JAK/STAT signaling pathway in ENKTL. Mutations affecting the C-terminal domain of SOCS1, including the SOCS box, result in the abnormal stabilization of JAK2 and the dysregulation of JAK/STAT signaling [103,104]. Receptor-type tyrosine-protein phosphatase κ (PTPRK) is a protein tyrosine phosphatase at chromosome 6q that contains a STAT3-specifying motif. The suppression of the expression of PTPRK by gene deletion and promotor hypermethylation leads to the activation of STAT3 [105]. PTPRK mutation was observed in 10% of ENKTLs [91].

- B. Epigenetic Modifiers

Epigenetic modifiers comprise the largest group among the mutated genes according to the NGS data reported to date (Figure 3). *MLLs* and *BCOR* are the most commonly mutated, followed by *TET2*, *EP300*, and *ARID1A* [85,86,87,88,89,90,91,92]. MLL2 (KMT2D) and MLL3 (KMT2C) belong to the KMT2 family, which methylates histone 3 lysine 4, which is associated with transcriptionally active chromatin [106]. KMT2 family enzymes play vital roles in a diverse set of cancers, both as drivers of oncogenesis and through critical cooperating mutations in both cancer progression and post-therapy relapse [106]. MLL2/KMT2D and MLL3/KMT2C are mutated in a significant percentage of malignant lymphomas of B- and T-cell lineage [107,108,109]. In ENKTL, MLL2/KMT2D and MLL3/KMT2C were found to be mutated in 58 of 443 cases (13.1%; range: 2–19%) [85,86,87,88,89,90,91] and in 19 of 125 cases (15.2%; range: 12–16%) [86,91]. The role of the MLL2/MLL3 complex in ENKTL is not clear. A recent study demonstrated that a mutation in the KMT2D gene was associated with the loss of its protein’s expression and poor survival in ENKTL, indicating a tumor-suppressing role [87]. The BCL6 corepressor (BCOR) is a transcriptional corepressor in association with BCL-6 and is also involved in histone modification when bound to polycomb repressive complex 1 [110,111,112]. BCOR is involved in normal hematopoiesis and lymphoid development [113,114,115]. Recurrent somatic clonal mutations of the BCOR gene and its homolog BCORL1 have been detected in several hematological malignancies, including myeloid dysplasia, acute myeloid leukemia, and B- and T-cell non-Hodgkin’s lymphomas [116]. The BCOR mutation was reported to be higher in EBV infection-associated malignancies, including EBV-positive gastric adenocarcinoma and ENKTL [90]. In ENKTL, BCOR was mutated in 42 of 370 cases examined by NGS, ranging from 6% to 32% [86,87,90,91,92,93,94]. The pattern of BCOR aberrations, including nonsense mutations, frame-shift mutations, mutations leading to splicing errors, and gene loss suggested a tumor-suppressor role for BCOR [86,116]. BCOR silencing significantly enhanced cell proliferation, AKT phosphorylation, and IL-2 production [117]. Other epigenetic modifiers, including TET2/EP300/ARID1A, have been found to be mutated less frequently than KMT family genes and BCOR. TET2 is frequently silenced by promotor hypermethylation in NKTCL [118]. The mutations of TET2 are mainly nonsense or small insertion–deletion mutations resulting in frameshifts, which leads to a loss of protein expression [87]; this was observed in 23 of 248 cases (9.3%) of ENKTL [86,87,91]. Somatic mutations of TET2 are associated with worse survival in ENKTL [87,119]. EP300 functions as a histone acetyltransferase, which regulates the transcription of genes via chromatin remodeling and is a major regulator of key signaling pathways [120]. EP300 is a critical regulator of hematopoiesis through both its transcriptional coactivator and acetyltransferase activities [121]. The mutations of EP300 in cancer encompass microdeletions, truncating mutations, and point mutations in different domains [120] and occurred in 30 of 379 ENKTL cases (7.9%) examined by NGS [87,88,91]. Stop-gain or frameshift mutations in ARID1A, leading to the loss of its expression, have been observed [122,123], and this occurred in 15 of 210 cases (7.1%) [85,88,91].

- C. Tumor Suppressor

In ENKTL, many tumor-suppressor genes are inactivated by promotor hypermethylation and deletion. TP53 mutations were detected in 105 of 242 cases (43.4%; range: 19–63%) by Sanger sequencing [18,80,81,82,124,125] and in 58 of 596 cases (9.7%; range: 4–25%) through NGS studies [85,86,87,88,89,90,91,92]. TP53 mutations predominantly affect the DNA-binding domain, and missense variants and stop-gain variants are the most common [126]. Clinically, TP53 mutations and p53 expression are associated with worse prognoses and higher disease stages [88,126]. MAX gene-associated protein (MGA), a suppressor of MYC, is frequently subject to loss-of-function mutations and copy-number deletions in multiple cancer types [127,128]. In the MYC/MAX/MGA pathway, MGA binds to genes bound by MYC and represses the function of MYC in protein interactions, transcriptional regulation, and cellular proliferation [127]. The molecular silencing of MGA resulted in decreased expression of MGA and increased MYC expression [91]. MGA mutations were observed in 17 of 205 ENKTL cases (8.3%) [88,91].

- D. RAS/MAPK Pathway

The Ras/mitogen-activated protein kinase (RAS/MAPK) pathway regulates cellular processes such as proliferation, survival, and migration [129]. The mutation and abnormal expression of genes involved in this pathway are major triggers for the development of most cancer types. Regarding the genes in the RAS/MAPK signaling pathway, RAS mutations are uncommon, while MAP3K5 and BRAF are consistently mutated in 10% and 8% of ENKTLs, respectively [91]. In melanoma, somatic mutations in MAP3K5 attenuate its proapoptotic function [130]. BRAF is a member of the RAF kinase family of growth signal transduction protein kinases, and the BRAF V600E mutation is an important genetic event in the tumorigenesis of multiple types of cancers, including malignant melanoma, thyroid cancer, hairy-cell leukemia, Langerhans-cell histiocytosis, and Erdheim–Chester disease, but it has not received much attention in the context of T-cell lymphoma, as it is rarely reported [131]. Among nine NGS studies, Xiong et al. reported BRAF mutations in 9 out of 100 ENKTL cases [91], while other studies did not report BRAF mutations. This discrepancy may be attributed to differences in the analytical platforms and the filtering criteria for the variants, and it would be interesting to confirm BRAF mutations in a future study. EPHA1 belongs to the ephrin receptor subfamily of the protein-tyrosine kinase family, and EPHs/ephrins are implicated in a variety of physiological processes and cancer. EphA kinases function as negative regulators of the RAS/MAPK pathway, exerting antimitogenic functions in a cell type-specific manner [132]. The activation of the EPHA receptor inhibits the TCR-induced activation of the RAS/MAPK pathway in thymocytes and anti-CD3-induced apoptosis [133]. EPHA1 mutation was observed in 13% of ENKTLs in one study, but its role in ENKTL is not clear [91].

- E. RNA Helicase

The RNA helicase family is one of a large group of genes altered in ENKTL, accounting for 8.4% of the mutated genes detected using an NGS platform [82,84,85,86,89,90]. The frequency was the highest in the study by Xiong et al., where mutations of the RNA helicase family, including DDX3X, DHX58, DDX21, and DDX18, were found in 44% of 100 ENKTLs [91]. Among the RNA helicase family, DDX3X is the most frequently mutated, as it was detected as mutated in 77 of 506 cases of ENKTL (15%; range: 8–50%) [84,86,87,88,91,92]. As in BCOR, DDX3X mutations are more common in EBV+ cancer [134]. Half of the alterations represented nonsense, splice-site, frameshift, and copy-loss mutations, which predominantly affect the helicase domains at amino acids and can lead to the truncation or loss of the DDX3X protein [88]. In addition, DDX3X silencing can be mediated by the significant promoter hypermethylation observed in ENKTL compared with normal NK cells [118]. Functionally, tumors with mutated DDX3X exhibit decreased RNA unwinding activity, a loss of suppressive function on cell-cycle progression, and the transcriptional activation of the NF-kB and MAPK pathways [88]. These findings indicate that DDX3X in ENKTL is a negative regulator of NK cell proliferation and mutation; disrupting this critical function contributes to ENKTL pathogenesis. In ENKTL, mutations of DDX3X and TP53 do not overlap, suggesting that the two genes are involved in closely related biological process and cooperate to function as tumor suppressors [88]. Clinically, Jiang et al. [88] demonstrated that patients with DDX3X mutations presented in higher subgroups regarding the international prognostic index, and individuals with mutations of DDX3X or TP53 had poor prognosis, but another study failed to validate the prognostic impact of DDX3X [91].

- F. Others

IKBKB and BIRC3, involved in the NF-kB pathway, are mutated at a low frequency, while the evolutionarily conserved signaling intermediate in Toll pathway (ECSIT) was subject to a hotspot mutation; ECSIT-V140A occurred in 17 of 88 (19%) ENKTLs and was associated with the activation of NF-kappa B, a higher incidence of hemophagocytic syndrome, and poor prognosis [92]. The evolutionarily conserved Notch signaling pathway regulates the differentiation and function of mature T lymphocytes [135]. Notch signaling pathway mutations were detected for NOTCH1 in 12 of 123 cases (10%) [87,91] and in 8 of 276 cases (3%) for NOTCH2 [84,87,91,92] in ENKTL. Although there are no functional data for Notch alterations in ENKTL, the MYC oncogene plays a major role in NOTCH1-induced transformation in T-ALL, and the oncogenic activity of NOTCH1 is strictly dependent on MYC upregulation [136]. Major histocompatibility complex (MHC) class II transactivator (CIITA) is the master regulator of MHC class II gene expression [137]. The mutation of CIITA was reported in 12 of 123 (10%) ENKTLs and is also frequently found in other types of malignant lymphoma, including primary mediastinal large B-cell lymphoma, follicular lymphoma, and Burkitt lymphoma [138,139,140]. The genomic aberrations of CIITA in B-cell lymphoma frequently comprise structural genomic rearrangements and missense, nonsense, and frameshift mutations, leading to a decreased expression of MHC-II genes and promoting the immune escape of the tumor [138].

## 7. Molecular Subtype of ENKTL

Molecular subtyping is a new way to classify cancers into different groups based on multi-omic data. Xiong J. et al. [91] performed a genomic and transcriptomic study on 128 biopsies of ENKTL and proposed three molecular subtypes: tumor suppressor–immune modulator (TSIM), MGA–BRDT (MB), and HDAC9–EP300–ARID1A (HEA). TSIM mainly involves the deletion of chromosome 6q21, containing tumor-suppressor genes; 9p24.1/PDL1/2 and JAK2 amplification; 17q21.2/STAT amplification; JAK/STAT pathway mutation; TP53 mutation; increased expression of NK cell-associated immunity; immune responses associated with antigen processing and presentation; and genomic instability. TSIM presented significantly higher NK gene expression, while HEA presented higher T-cell gene expression. When the EBV transcript was compared with the molecular subtypes, TSIM presented a latency II pattern and higher level of the lytic gene BALF3, which is associated with DNA damage and genomic instability [141]. MB is characterized by LOH in 1p22.1/BRDT and MGA mutations related to the upregulation of MYC, and the MAPK, Notch, and WNT signaling pathways. MB showed a latency I pattern. Clinically, MB has a poor prognosis compared to the TSIM and HEA subtypes. HEA is characterized by the mutation of epigenetic modifiers with the activation of the NF-kB pathway and TCR signaling pathway. HEA presents a latency II pattern and a higher level of the lytic gene BNRF1, which is important for the establishment of latency and cell immortalization [142]. When considering these data, the TSIM molecular subgroup appears to represent the prototype of ENKTL, which is typically of the NK lineage, having deletions or mutations of many tumor-suppressor genes and having mutations of genes in the JAK/STAT pathway, as well as a high expression of PDL1 in tumor tissue. Alternatively, the HEA subtype appears to correspond to ENKTL of T-cell lineage, which has few pathological and clinical differences from ENKTL of NK cell lineage. The MB subtype represents the worst prognostic group, with an increased expression of MYC resulting from a silencing mutation of MGA.

## 8. Genetic Alteration According to the Cell of Origin

The tumor cells of ENKTL consist of either NKs or cytotoxic T cells. ENKTL has few pathological and clinical differences according to cell lineage. The mutational landscapes of NK and T-cell-derived ENKTLs showed no difference in a study by Jiang L. et al. analyzing 105 cases of ENKTL [88]. However, a study by Xiong J. et al. [91] demonstrated clear differences in the mutation landscapes between NK and T-cell lineage ENKTLs. NK cell lineage ENKTL presented frequent mutations/CNVs of STAT3, DDX3X, KMT2C, JAK2, KMT2D, EP300, STAT5B, and STAT5A, while tumors of T-cell lineage presented variations involving EPHA1, TP53, ARID1A, PTPRQ, NCOR2, PPFIA2, BCOR, PTPRK, and HDAC, suggesting that the JAK/STAT signaling pathway plays a key role in ENKTLs of NK lineage, while the disruption of the RAS-MAPK signaling pathway and epigenetic modifiers is the main mechanism of the tumorigenesis of ENKTLs of T-cell lineage [91].

## 9. Genetic Alterations of EBV Infected in ENKTL

Genetic alterations are crucial for the pathogenesis of ENKTL, but epigenetic alterations may be more important during EBV-associated tumorigenesis [143]. EBV plays a key role as an epigenetic driver in EBV-associated cancer. EBV-encoded oncoproteins including LMP1, LMP2, and EBNA3 modulate host-cell epigenetic machinery, reprograming the viral and host epigenomes, using host epigenetic modifiers, including DNA methyltransferases, histone methyltransferases, polycomb group proteins, and histone deacetylases [144,145,146]. In addition, EBV-encoded miRNAs are another epigenetic regulatory mechanism and regulate host-cell biology and the microenvironment, contributing to the cell proliferation, migration, and immune evasion of EBV-infected cells [147,148]. Chakravorty P. et al. constructed a comprehensive host–virus interactome map using a publicly available database for EBV-positive cancers, including 18 cases of ENKTL [134]. Across the different cancers, DDX3X, MYC, BCOR, ARID1A, TRAF3, EP300, PTEN, CASP8, TP53, and ID3 were more frequently mutated in EBV+ cancer [134]. The majority of EBV genomes exist as nonintegrated episomes in the nucleus with a nucleosomal pattern similar to that of host chromatin [149] but can also integrate into the cancer genome [150,151]. Specific integration loci include B2M, CD74, and HLA-C, which are part of MHC class I and II complexes. The top three pathways enriched in integration loci are a viral process, response to type I IFNs, and the regulation of the apoptotic pathway in response to DNA damage [134]. Peng et al. [151] analyzed the genomic and transcriptomic characteristics of EBV, which infected tumor cells in 27 cases of ENKTL. The EBV sequence accounts on average for 0.45% (0.03–1.06%) of the whole-genome sequencing data. Mutational hotspots were found at the BPLF1 and BDLF2/3 regions, in addition to those at EBNAs, LMP1, and LMP2 that are consistently reported in EBV derived from other cancers. Mutations of the T-cell epitopes of EBV genes were frequent, suggesting a distinct mechanism for immune evasion. A 30 bp deletion at LMP1 and small deletions at EBNA2, BLLF1/2, EBNA3s, and LMP1 were frequent [151].

## 10. Genetic Alterations of ENKTL in Comparison with Aggressive NK Cell Leukemia, EBV-Positive Nodal T-/NK Cell Lymphoma, and Large Granular Lymphocytic Leukemia

Extranodal NK/T-cell lymphoma and aggressive NK cell leukemia (ANKL) are the prototypes of EBV-positive T- or NK cell malignancy, with distinct clinical findings. EBV-positive nodal T- or, uncommonly, NK cell lymphoma is a recently recognized tumor characterized by an EBV-positive tumor present in the lymph nodes. All of these three entities share EBV as an important etiologic factor and a cytotoxic phenotype of either T or NK cells, but they are different in clinical presentation.

An early aCGH study by Nakashima Y. et al. [69] reported differences in genomic alterations between ENKTL and ANKL. ENKTL showed a recurrent gain of 2q33.1–q37.3 containing WNT6; gain of 17q21.2–q21.31 containing STAT3/5B/5A; loss of 1p36.23–p36.33 containing PRDM1; loss of 2p16.1–p16.3 containing BIN1; loss of 4q12 containing KIT; loss of 4q31.3–q32.1 containing PDGFC; and loss of 5p14.1–p14.3, 5q34–q35.3, and 6q21–q22.1 containing FOXO3, HACE1, PRDM1, ATG5, and AIM1, while aggressive NK cell leukemia showed more frequent gains of 1q23.1–q24.2 and 1q31.3–q44, as well as losses of 7p15.1–p22.3 and 17p13.1 containing TP53.

In the NGS era, data from three NGS studies showed more similarities than differences between the two entities. Hussein E. et al. [59] analyzed eight cases of ANKL and observed the recurrent mutations of genes involved in DNA damage, including TP53, ASXL1, and ASXL2, and the epigenetic regulator TET2. The integrated genomic or transcriptomic and functional analysis of 29 cases of ANKL by Huang et al. [152] demonstrated that the JAK/STAT signaling pathway is the major target of genetic alterations leading to the activation of STAT3 and the increased expression of MYC. Additional frequent mutations in their study involved TP53, TET2, CREBBP, and MLL2. BCOR and DDX3X, which are frequently mutated in ENKTL were mutated at low frequency. Dufva O. et al. [153] investigated 14 ANKL cases by whole-exome sequencing and identified mutations in STAT3 (21%), RAS-MAPK pathway genes (21%), DDX3X (29%), and epigenetic modifiers (50%). Additional alterations include JAK/STAT copy gains and tyrosine-phosphatase mutations. Taken together, those NGS data suggest that ENKTL and ANKL share a common molecular pathway in lymphomagenesis.

EBV-positive nodal T-cell lymphoma is a tumor mainly derived from cytotoxic T cells expressing alpha–beta or gamma–delta receptors or lacking TCR antigen expression [54,55,154]. Little is known about the genetic alterations in this tumor. A gene-expression-profiling assay by Ha S. et al. reported the overexpression of immune-response genes, suggesting a specific relation between EBV infection and the alteration of the immune response in patients with EBV-positive nodal T-cell lymphoma [154]. A study using microarray and aCGH analysis by Ng SB et al. [155] demonstrated EBV-positive nodal T-cell lymphoma enriched in the genes involved in MTOR signaling and IL6/JAK/STAT3 signaling, as well as several genes involved in the cell cycle and genomic instability. In their study, the copy-number profile revealed similarities and differences between ENKTL and EBV-positive nodal T-cell lymphoma. Distinct from ENKTL is 14q11.2 loss of EBV-positive nodal T-cell lymphoma, which correlates with the loss of TCR loci and a T-cell origin [155]. Future genetic studies of this rare tumor are needed to elucidate its molecular pathogenesis.

Chronic lymphoproliferative disorder of NK cells (CLPD-NK) and T-cell large granular lymphocytic leukemia (T-LGL) are chronic clonal lymphoproliferative disorders with similar indolent clinical features and accumulation of cytotoxic granular lymphocytes of either NK or T-cell lineage [156]. Recent studies revealed that they share frequent mutation of STAT3 involving the SH domain found in 27–47.6% of T-LGL and 27.2–70% of CLPD-NK [156,157,158]. Although CLPD-NK and T-LGL are recognized as a distinct entities based on cell lineage, similar prevalence of STAT3 mutation suggests common pathogenesis sharing at least in a subset of cases between the two entities [156]. More recently, a damaging mutation of TET2 was detected in 28–34% of NK cell compartment of CLPD-NK with co-mutations of TET2 and STAT3 in 2–12% of cases [159,160]. In addition to mutation, TET2 genes in CLPD-NK are frequently affected by promotor methylation. These cases with dysfunctional TET2 exhibit enhanced global methylation, and the TET2-mutated CLPD-NK was enriched in hypermethylation of PTPRD and PTPRN which are negative regulators of STAT3 [160]. These findings suggest that, in addition to the STAT3 mutation, epigenetic dysregulation of the JAK/STAT pathway by TET2 is an important molecular mechanism of CLPD-NK oncogenesis.

ENKTL is distinct from CLPD-NK and T-LGL in clinical aspects and the presence of EBV in tumor cells but shares a high frequency of STAT3 mutation. Epigenetic modifiers in ENKTL were the largest group affected by the mutations. Although the action mechanism and target genes of epigenetic modifiers have not yet been well elucidated, epigenetics are highly likely to play a central role in tumorigenesis of ENKTL, as demonstrated in TET2 and STAT3 of CLPD-NK.

## 11. Conclusions

It has been more than 130 years since ENKTL was first described as “ulceronecrotic proliferative lesions of the upper airways”. Since the conception of the disease, it has been established that this tumor mainly consists of NK cells and that EBV infects most of the tumor cells. The recently developed molecular genetic analysis technology allows for exploring the genetic and epigenetic characteristics of ENKTL, as well as the mechanism by which EBV infection in NK cells is involved in tumorigenesis. However, data on the functions and interactions of these genes in the lymphomagenesis of ENKTL are very scarce. More studies in this field would be very helpful in better understanding the molecular genetic pathogenesis of ENKTL and establishing better treatment strategies.

## Figures and Tables

**Table 1:** Other EBV-positive NK/T-cell malignancies of adult.

	ANKL	NTNKL	IVNKTL
Sex	Male = female	Male > female	Male > female
Median age	40 years	62 years	46 years
Ethnicity	Asian	Asian	Asian and Latin American
Localization	PB, BM, liver, spleen	Systemic nodal	Intravascular growth
Pathologic characteristics	Leukemic cells, from normal to highly atypical features CD3+, CD56+, cytotoxic molecules+, EBER+	High-grade morphology with centroblastoid appearance CD3+, CD8+, CD56−, cytotoxic molecules+, EBER+	Intraluminal large atypical cells CD3+, CD56+, cytotoxic molecules+, EBER+
Clinical course	Aggressive	Aggressive	Aggressive

ANKL, aggressive NK cell leukemia; NTNKL, nodal T-/NK cell lymphoma; IVNKTL, intravascular NK/T-cell lymphoma; PB, peripheral blood; BM, bone marrow.

## Data Availability

No new data were created or analyzed in this study.

## Supplementary Materials

The following supporting information can be downloaded at: www.mdpi.com/article/10.3390/life12010073/s1, Table S1: Frequency of somatic gene mutations found by next generation sequencing in extranodal NK/T cell lymphoma.
